# Supplementary material for: Pain persists in mice lacking both Substance P and CGRPα signaling
Source: bioRxiv. 2024 Dec 2:2023.11.15.567208. Originally published 2023 Nov 17. Preprint. [Version 2] doi: 10.1101/2023.11.15.567208 (PMC10705526; doi:10.1101/2023.11.15.567208)
Supplement: Supplement 1 [file NIHPP2023.11.15.567208v2-supplement-1.pdf]

bioRxiv preprint doi: https://doi.org/10.1101/2023.11.15.567208; this version posted December 2, 2024. The copyright holder for this preprint (which was not certified by peer review) is the author/funder. This article is a US Government work. It is not subject to copyright under 17 USC 105 and is also made available for use under a CC0 license.

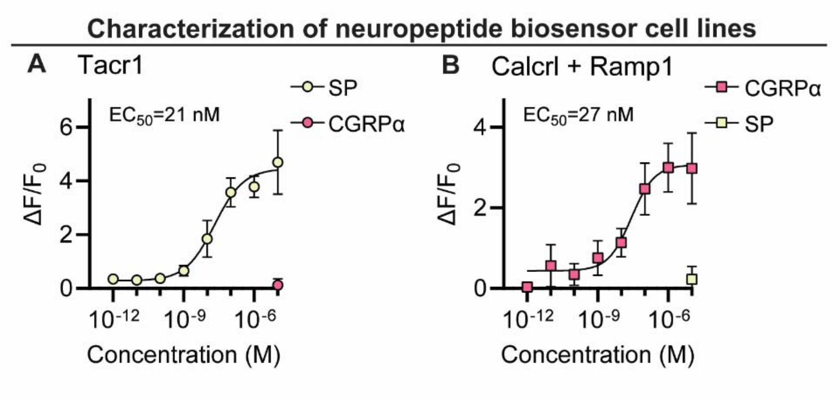

Figure 1 – figure supplement 1. Substance P-sniffer cells selectively respond to Substance P. (A) Dose-response curve showing Substance P activates Substance P-sniffer cells at low nanomolar concentrations (EC_{50}=11.8 nM), but cells are insensitive to CGRPα. **(B)** Dose-response curve showing CGRPα activates CGRP-sniffer cells at low nanomolar concentrations (EC_{50}=11.8 nM), but cells are insensitive to Substance P. 4-parameter variable slope dose-response curves were fit by non-linear regression. At least three replicates were performed for each concentration per condition, in two independent experiments.

bioRxiv preprint doi: https://doi.org/10.1101/2023.11.15.567208; this version posted December 2, 2024. The copyright holder for this preprint (which was not certified by peer review) is the author/funder. This article is a US Government work. It is not subject to copyright under 17 USC 105 and is also made available for use under a CC0 license.

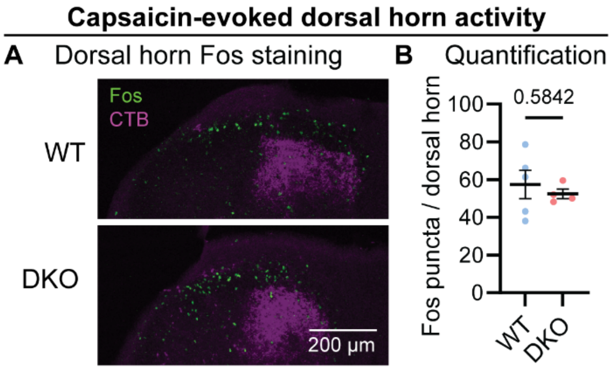

Figure 2 – figure supplement 1. Capsaicin evokes Fos activity in the dorsal horn of Tac1::Calca DKO mice. (A) Example confocal images showing dorsal horn of WT and DKO mice backlabelled with CTB (*magenta*) from the paw. Similar numbers of Fos puncta (*green*) are visible in the ipsilateral superficial dorsal horn of the WT and DKO cases. **(B)** Quantification of the mean number of Fos puncta in the ipsilateral dorsal horn of WT and DKO mice. For each mouse, the number of Fos puncta was counted in the 5 sections with the strongest CTB labelling and then averaged so that *n* is the number of mice. *n*=5 (2M, 3F) for WT & *n*=4 (2M, 2F) for DKO. Means were compared for (B) using an unpaired t test. Error bars denote standard error of the mean.

bioRxiv preprint doi: https://doi.org/10.1101/2023.11.15.567208; this version posted December 2, 2024. The copyright holder for this preprint (which was not certified by peer review) is the author/funder. This article is a US Government work. It is not subject to copyright under 17 USC 105 and is also made available for use under a CC0 license.

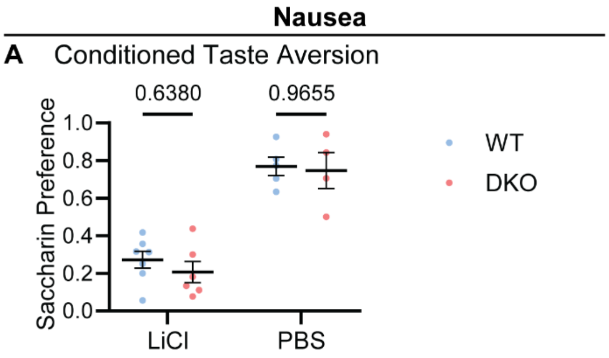

Figure 2 – figure supplement 2. Tac1::Calca DKO mice develop LiCl-induced conditioned taste aversion (A) Quantification of conditioned taste aversion (CTA) test in WT (blue) and DKO (red) mice. Saccharin preference index on test day is shown for animals given either lithium chloride or PBS following saccharin exposure on the conditioning day. Both WT and DKO treated with LiCl show a pronounced aversion to the usually-preferred saccharin. For LiCl, *n*=7 (4M, 3F) for WT & *n*=6 (4M, 2F) for DKO. For PBS, *n*=5 (3M, 2F) for WT & *n*=4 (2M, 2F) for DKO. Means were compared by 2-way ANOVA followed by post-hoc Sidak's test. Error bars denote standard error of the mean.

Supplementary Table 1. Summary of statistical tests.

Figure	Name	Variable (units)	N	Passed Shapiro-Wilk Test for Normality?	Test	Comparison	Statistic	P	Post hoc analysis	Comparison	P (adjusted)
**1E**	Substance P Release	NKR1 Activation (deltaF/F0)	WT: 12; DKO: 12		2-WAY RM ANOVA	Interaction	F (2, 44) = 3.718	0.0322	Sidak's multiple comparisons test	Vehicle - WT vs DKO	0.86
						Drug	F (1.569, 34.51) = 78.37	<0.0001		Capsaicin - WT vs DKO	0.013
						Genotype	F (1, 22) = 5.226	0.0322		SP - WT vs DKO	0.893
**1H**	CGRP Release	CLR Activation (deltaF/F0)	WT: 18; DKO: 18		2-WAY RM ANOVA	Interaction	F (2, 68) = 5.317	0.0072	Sidak's multiple comparisons test	Vehicle - WT vs DKO	0.499
						Drug	F (1.106, 37.61) = 22.49	0.0001		Capsaicin - WT vs DKO	2E-04
						Genotype	F (1, 34) = 0.006702	0.9352		CGRP - WT vs DKO	0.421
**2A**	von Frey	50% Threshold (log g)	WT: 14; DKO: 14	Yes (WT: W=0.9238, P=0.2495; DKO: W=0.9521, P=0.5943)	Unpaired t-test	WT vs DKO	t=1.007	0.3231			
**2B**	Pinprick	Response (%)	WT: 13; DKO: 11	No (WT: W=0.813, P=0.0098; DKO: W=0.7538, P=0.0023)	Mann Whitney U Test	WT vs DKO	U=59.5	0.5499			
**2C**	Clip	Attending (s)	WT: 7; DKO: 10	Yes (WT: W=0.8768, P=0.2127; DKO: W=0.9484, P=0.6502)	Unpaired t-test	WT vs DKO	t=0.6762	0.5092			
**2D**	Hargreaves	Latency (s)	Low - WT: 12; DKO: 8; High - WT: 15; DKO: 17		2-WAY ANOVA	Interaction	F (1, 48) = 0.3387	0.5633	Sidak's multiple comparisons test	Low - WT vs DKO	0.651
						Intensity	F (1, 48) = 9.597	0.0033		High - WT vs DKO	0.991
						Genotype	F (1, 48) = 0.5373	0.4671			
**2E**	Hot Plate	Latency (s)	52.5 - WT: 15; DKO: 10; 55.5 - WT: 9; DKO: 8		2-WAY ANOVA	Interaction	F (1, 38) = 0.03579	0.851	Sidak's multiple comparisons test	52.5 - WT vs DKO	0.987
						Intensity	F (1, 38) = 14.27	0.0005		55.5 - WT vs DKO	0.918
						Genotype	F (1, 38) = 0.1417	0.7087			
**2F**	Acetone	Licking (s)	WT: 12; DKO: 12	Yes (WT: W=0.9097, P=0.2113; DKO: W=9371, P=0.4616)		WT vs DKO	t=0.3759	0.7106			
**2G**	Dry Ice	Latency (s)	WT: 19; DKO: 13	No (WT: W=0.9051, P=0.0602; DKO: W=0.7896, P=0.0051)	Mann Whitney U Test	WT vs DKO	U=111	0.643			
**2H**	Capsaicin	Licking (s)	WT: 9; DKO: 8	Yes (WT: W=0.926, P=0.4439; DKO: W=0.9062, P=0.3278)		WT vs DKO	t=2.041	0.0593			

bioRxiv preprint doi: https://doi.org/10.1101/2023.11.15.567208; this version posted December 2, 2024. The copyright holder for this preprint (which was not certified by peer review) is the author/funder. This article is a US Government work. It is not subject to copyright under 17 USC 105 and is also made available for use under a CC0 license.

Figure	Name	Variable (units)	N	Passed Shapiro-Wilk Test for Normality?	Test	Comparison	Statistic	P	Post hoc analysis	Comparison	P (adjusted)
**2I**	AITC	Licking (s)	WT: 12; DKO: 12	No (WT: W=0.8549, P=0.0422; DKO: W=0.8314, P=0.0218)		WT vs DKO	U=44	0.1135			
**2J**	Acetic Acid	Writhes (#)	WT: 9; DKO: 7	Yes (WT: W=0.9108, P=0.3214; DKO: W=9211, P=0.4778)		WT vs DKO	t=0.3757	0.7127			
**2K**	Chloroquine	Scratching Bouts (#)	WT: 8; DKO: 11	No (WT: W=0.9211, P=0.4392; DKO: W=0.7614, P=0.0029)			U=39	0.7168			
**2-S1**	Capsaicin Fos	Fos puncta / dorsal horn (#)	WT: 5; DKO: 4	Yes (WT: W=0.9444, P=0.6974; DKO: W=0.8844, P=0.3597)			t=0.5736	0.5842			
**2-S2**	Conditioned Taste Aversion	Latency (s)	LiCl- WT: 7; DKO: 6; PBS - WT: 5; DKO: 4		2-WAY ANOVA	Interaction	F (1, 18) = 0.1361	0.7165	Sidak's multiple comparisons test	LiCl	0.638
						Intensity	F (1, 18) = 75.79	<0.0001		PBS	0.966
						Genotype	F (1, 18) = 0.5413	0.4714			
**3A**	CFA -- Hargreaves	Latency (s)	WT: 9; DKO: 10		2-WAY RM ANOVA	Interaction	F (3, 51) = 0.4715	0.7035	Sidak's multiple comparisons test	0d - WT v DKO	>0.9999
						Time	F (2.117, 35.99) = 17.63	<0.0001		1d - WT v DKO	0.996
						Genotype	F (1, 17) = 0.8594	0.3669		2d - WT v DKO	0.334
										7d - WT v DKO	0.734
**3B**	CFA - von Frey	Threshold (log g)	WT: 6; DKO: 6		2-WAY RM ANOVA	Interaction	F (3, 30) = 0.07568	0.9726	Sidak's multiple comparisons test	0d - WT v DKO	0.998
						Time	F (3, 30) = 50.74	<0.0001		1d - WT v DKO	>0.9999
						Genotype	F (1, 10) = 0.02549	0.8763		2d - WT v DKO	>0.9999
										7d - WT v DKO	0.989
**3C**	PGE2 - Hargreaves	Latency (s)	WT: 10; DKO: 10		2-WAY RM ANOVA	Interaction	F (5, 90) = 2.053	0.0787	Sidak's multiple comparisons test	0m - WT vs DKO	0.738
						Time	F (5, 90) = 35.65	0.0001		15m - WT vs DKO	>0.9999
						Genotype	F (1, 18) = 3.020	0.0993		30m - WT vs DKO	>0.9999
										45m - WT vs DKO	0.765
										60m - WT vs DKO	0.804
										120m - WT vs DKO	0.025

bioRxiv preprint doi: https://doi.org/10.1101/2023.11.15.567208; this version posted December 2, 2024. The copyright holder for this preprint (which was not certified by peer review) is the author/funder. This article is a US Government work. It is not subject to copyright under 17 USC 105 and is also made available for use under a CC0 license.

Figure	Name	Variable (units)	N	Passed Shapiro-Wilk Test for Normality?	Test	Comparison	Statistic	P	Post hoc analysis	Comparison	P (adjusted)
**3D**	PGE2 - von Frey	Threshold (log g)	WT: 6; DKO: 6		2-WAY RM ANOVA	Interaction	F (4, 40) = 0.3472	0.8444	Sidak's multiple comparisons test	0m - WT vs DKO	0.856
						Time	F (2.803, 28.03) = 28.64	<0.0001		15m - WT vs DKO	0.977
						Genotype	F (1, 10) = 1.475	0.2525		30m - WT vs DKO	0.999
										45m - WT vs DKO	0.999
										60m - WT vs DKO	0.9
										120m - WT vs DKO	0.025
**3F**	Capsaicin - Oedema	Swelling (norm.)	WT: 9; DKO: 7	Yes (WT: W=0.9684, P=0.8811; DKO: W=0.8609, P=0.1541)	Unpaired t-test	WT vs DKO	t=0.8876	0.3897			
**3G**	Capsaicin - Extravasation	Optical Density (norm.)	WT: 9; DKO: 7	Yes (WT: W=0.9829, P=0.9776; DKO: W=0.8732, P=0.1981)	Unpaired t-test	WT vs DKO	t=0.2268	0.8239			
**3I**	AITC - Oedema	Swelling (norm.)	WT: 8; DKO: 8	Yes (WT: W=0.9428, P=0.639; DKO: W=0.8994, P=0.2853)	Unpaired t-test	WT vs DKO	t=0.05078	0.9602			
**3J**	AITC - Extravasation	Optical Density (norm.)	WT: 8; DKO: 8	Yes (WT: W=0.9459, P=0.6697; DKO: W=0.9597, P=0.8076)	Unpaired t-test	WT vs DKO	t=0.5681	0.579			
**4A**	SNI - Tactile Allodynia	Threshold (log g)	WT: 8; DKO: 8		2-WAY RM ANOVA	Interaction	F (5, 70) = 1.161	P=0.3370	Sidak's multiple comparisons test	0d - WT v DKO	0.952
						Time	F (5, 70) = 46.15	P<0.0001		2d - WT v DKO	0.731
						Genotype	F (1, 14) = 0.01651	P=0.8996		5d - WT v DKO	1
										7d - WT v DKO	>0.9999
										14d - WT v DKO	0.753
										21d - WT v DKO	0.991
**4C**	SNI - Fos staining	Fos puncta / dorsal horn (#)	WT: 4; DKO: 3		2-WAY ANOVA	Interaction	F (1, 5) = 0.02824	0.8731	Sidak's multiple comparisons test	Ipsi - WT vs DKO	0.815
						Side	F (1, 5) = 18.60	0.0076		Contra - WT vs DKO	0.922
						Genotype	F (1, 5) = 0.3993	0.5552			
**4D**	Oxaliplatin - Cold Plate	Behaviors (s)	WT: 9; DKO: 3	Yes (WT: W=0.9402, P=0.5843; DKO: W=0.9756, P=0.9274)	Unpaired t-test	WT vs DKO	t=0.8884	0.3905			

bioRxiv preprint doi: https://doi.org/10.1101/2023.11.15.567208; this version posted December 2, 2024. The copyright holder for this preprint (which was not certified by peer review) is the author/funder. This article is a US Government work. It is not subject to copyright under 17 USC 105 and is also made available for use under a CC0 license.
