# Supplementary material for: Pain persists in mice lacking both Substance P and CGRPα signaling
Source: bioRxiv. 2023 Nov 17:2023.11.15.567208. Preprint. [Version 1] doi: 10.1101/2023.11.15.567208 (PMC10705526; doi:10.1101/2023.11.15.567208)
Supplement: Supplementary file 1 [file NIHPP2023.11.15.567208V1-supplement-1.pdf]

bioRxiv preprint doi: https://doi.org/10.1101/2023.11.15.567208; this version posted November 17, 2023. The copyright holder for this preprint (which was not certified by peer review) is the author/funder. This article is a US Government work. It is not subject to copyright under 17 USC 105 and is also made available for use under a CC0 license.

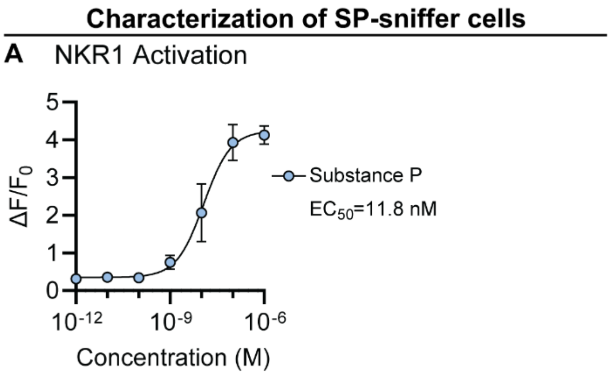

Figure 1 – figure supplement 1. Substance P-sniffer cells selectively respond to Substance P in an NKR1-dependent manner. (A) Dose-response curve showing Substance P activates Substance P-sniffer cells at low nanomolar concentrations (grey, EC_{50}=11.8 nM). 4-parameter variable slope dose-response curves were fit by non-linear regression. At least three replicates were performed for each concentration per condition, in two independent experiments.

bioRxiv preprint doi: https://doi.org/10.1101/2023.11.15.567208; this version posted November 17, 2023. The copyright holder for this preprint (which was not certified by peer review) is the author/funder. This article is a US Government work. It is not subject to copyright under 17 USC 105 and is also made available for use under a CC0 license.

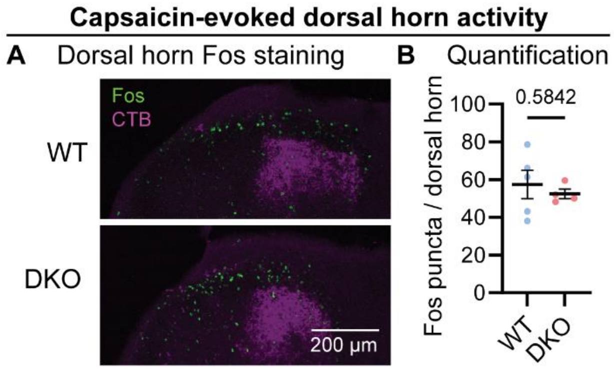

Figure 2 – figure supplement 1. Capsaicin evokes Fos activity in the dorsal horn of Tac1::Calca DKO mice. (A) Example confocal images showing dorsal horn of WT and DKO mice backlabelled with CTB (*magenta*) from the paw. Similar numbers of Fos puncta (*green*) are visible in the ipsilateral superficial dorsal horn of the WT and DKO cases. **(B)** Quantification of the mean number of Fos puncta in the ipsilateral dorsal horn of WT and DKO mice. For each mouse, the number of Fos puncta was counted in the 5 sections with the strongest CTB labelling and then averaged so that *n* is the number of mice. *n*=5 (2M, 3F) for WT & *n*=4 (2M, 2F) for DKO. Means were compared for (B) using an unpaired t test. Error bars denote standard error of the mean.

bioRxiv preprint doi: https://doi.org/10.1101/2023.11.15.567208; this version posted November 17, 2023. The copyright holder for this preprint (which was not certified by peer review) is the author/funder. This article is a US Government work. It is not subject to copyright under 17 USC 105 and is also made available for use under a CC0 license.

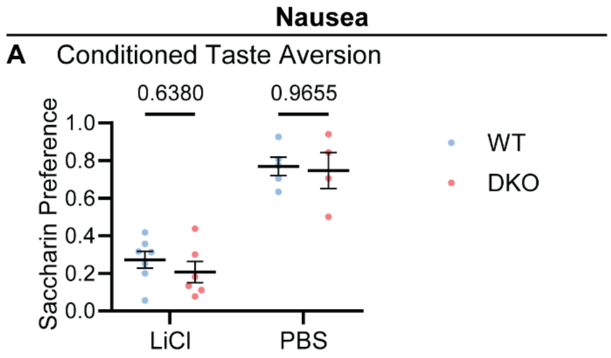

Figure 2 – figure supplement 2. Tac1::Calca DKO mice develop LiCl-induced conditioned taste aversion (A) Quantification of conditioned taste aversion (CTA) test in WT (blue) and DKO (red) mice. Saccharin preference index on test day is shown for animals given either lithium chloride or PBS following saccharin exposure on the conditioning day. Both WT and DKO treated with LiCl show a pronounced aversion to the usually-preferred saccharin. For LiCl, n=7 (4M, 3F) for WT & n=6 (4M, 2F) for DKO. For PBS, n=5 (3M, 2F) for WT & n=4 (2M, 2F) for DKO. Means were compared by 2-way ANOVA followed by post-hoc Sidak's test. Error bars denote standard error of the mean.
